# Towards Multi-Organoid Systems for Drug Screening Applications

**DOI:** 10.3390/bioengineering5030049

**Published:** 2018-06-21

**Authors:** Cláudia C. Miranda, Tiago G. Fernandes, Maria Margarida Diogo, Joaquim M. S. Cabral

**Affiliations:** 1 iBB-Institute for Bioengineering and Biosciences and Department of Bioengineering, Instituto Superior Técnico, Universidade de Lisboa, Av. Rovisco Pais, 1049-001 Lisbon, Portugal; claudia.miranda@tecnico.ulisboa.pt (C.C.M.); tfernandes@tecnico.ulisboa.pt (T.G.F.); margarida.diogo@tecnico.ulisboa.pt (M.M.D.); 2 The Discoveries Centre for Regenerative and Precision Medicine, Lisbon Campus, Instituto Superior Técnico, Universidade de Lisboa, Av. Rovisco Pais, 1049-001 Lisbon, Portugal

**Keywords:** human pluripotent stem cells, organoids, body-on-a-chip, multi-organ systems, drug discovery, personalized medicine

## Abstract

A low percentage of novel drug candidates succeed and reach the end of the drug discovery pipeline, mainly due to poor initial screening and assessment of the effects of the drug and its metabolites over various tissues in the human body. For that, emerging technologies involving the production of organoids from human pluripotent stem cells (hPSCs) and the use of organ-on-a-chip devices are showing great promise for developing a more reliable, rapid and cost-effective drug discovery process when compared with the current use of animal models. In particular, the possibility of virtually obtaining any type of cell within the human body, in combination with the ability to create patient-specific tissues using human induced pluripotent stem cells (hiPSCs), broadens the horizons in the fields of drug discovery and personalized medicine. In this review, we address the current progress and challenges related to the process of obtaining organoids from different cell lineages emerging from hPSCs, as well as how to create devices that will allow a precise examination of the in vitro effects generated by potential drugs in different organ systems.

## 1. Introduction

Presently, only 10% of drugs that enter Phase I of clinical trials will succeed [1]. This emphasizes the need to develop and optimize methodologies that will reduce the expensive costs and overall time needed to identify a suitable drug candidate (Table 1). Current methods involve the use of two-dimensional (2D) cell cultures, most often immortalized cell lines, which often fail to reproduce cell-cell interactions and cell morphology in native microenvironments. Moreover, the use of animal models, often adopted in drug discovery applications, may contain interspecies differences, which can result in drug failure or unanticipated side-effects upon translation to humans, further increasing the time and costs of new drug candidate development. Importantly, toxic effects in secondary tissues can be as significant as effects on the target site and mimicking such complex dynamics can be fundamental in the identification of suitable and safe drug candidates. However, existing models generally do not account for these effects.

**Figure 1:**
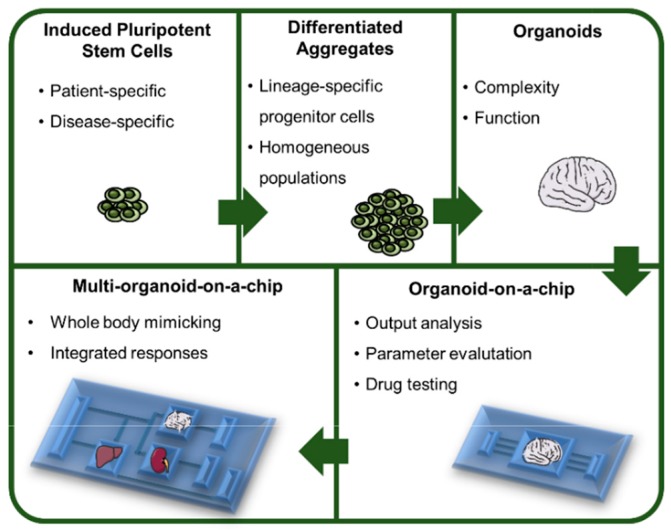
From human pluripotent stem cells (hPSCs) to multi-organoid platforms. The use of hiPSCs brings the possibility to generate patient- and disease-specific organoids that will contribute to advances in the fields of personalized medicine and disease modeling. These cells can be first differentiated into lineage specific progenitor cells that in turn will be able to generate organoids representing the complex interactions that exist between cells in vivo. The integration of these organoids within a culture/analysis chip provides the necessary tools to analyze different parameters that may be altered in specific conditions, such as exposure to a drug. Moreover, a chip that contains different types of organoids fulfills the necessity of analyzing the systems as a whole, considering the different interactions between organoids and providing an integrated response to a specific stimulus.

Currently, different organ-on-a-chip platforms have also been developed in which cells are cultured as three-dimensional (3D) aggregates that represent human tissue organization and function, and that are traditionally incorporated within a microfluidic device [2,3,4]. However, these systems still present some limitations and drawbacks, which hamper their full potential. First, they still lack several characteristics of normal human tissues, such as interactions with supportive cells and the presence of matrix proteins that allow the recapitulation of the in vivo environment. Second, although current reports on tissue model generation use sophisticated culture technologies such as patterned surfaces or three-dimensional (3D) bioprinting for tissue or organ construction [3,5], they mostly rely on the use of primary cells, which can constrain the natural self-organizing capacity of developing tissues. Therefore, the generation of organoids from human induced pluripotent stem cells (hiPSCs), mimicking important structural and functional aspects of human tissues in a 3D configuration, and potentially their integration with organ-on-a-chip devices can be advantageous for a more realistic modeling of both healthy and diseased tissues (Figure 1). The main features of hiPSCs include the capacity for self-renewal and proliferation, as well as their ability to differentiate into cells and tissues originated from the three embryonic germ layers. In fact, in the embryo, patterning signals are released from special signaling centers, regions that have strong inductive effects over a wide area of tissues, and that are responsible for the creation of morphogen gradients [6]. The same occurs in human pluripotent stem cells (hPSCs), which highlights the ability of the cells to regulate self-organization processes [7]. hiPSCs also provide the capacity to generate patient-specific cells that allow disease modeling, development of personalized medicine approaches and bypassing rejection in cell therapy settings [8,9,10].

Importantly, however, one major hurdle concerning hPSC commitment as 3D aggregates is the heterogeneity and disorganization that arises due to uncontrolled aggregation of cells that, in turn, leads to poor efficiencies of differentiation. In fact, the initial diameter of the hPSC aggregates will determine the overall efficiency of the differentiation process, as intrinsic signaling for each lineage specification is different [11]. For instance, it has already been demonstrated that cardiac and neural lineage differentiation efficiencies can be maximized by applying this principle, leading to an average 80% efficiency in neural induction [11,12]. Therefore, control over aggregate diameter may be able to address this challenge, providing adequate and homogeneous cues within the aggregate and increasing culture uniformity [13].

In this article, we review the state of the art regarding hPSCs differentiation from a pluripotent state to lineage committed spheroids, and further production of self-organized, functional organoids. We also highlight examples of organ-on-a-chip devices that have been used for testing the effect of drugs on organoids representing tissues such as heart and liver. We anticipate that different types of hPSC-derived organoids can be cultured in these devices and be used to model the interactions between different human organs allowing the prediction of the effects of multiple drugs when administered in the human body.

## 2. Controlled Differentiation of Human Pluripotent Stem Cells as 3D Aggregates

Current technologies involving the use of hPSCs offer the unlimited possibility of generating any type of cell within the human body. However, for many applications, there is still a lack of efficient methodologies to convert pluripotent stem cells into lineage-specific progenitors or into fully differentiated cells at a scale that is compliant with clinically relevant cell numbers [14]. Furthermore, processes to fully purify the final product before clinical applications should also be applied, including microfluidic separation, magnetic and fluorescent activated cell sorting or small molecules that selectively deplete remaining hPSCs from the final product [15,16].

Although 2D culture is able to efficiently sustain the growth of almost all cell types, it presents several limitations concerning the mimicking of in vivo conditions, namely, the ones related to cell to cell interactions and cell interactions with the extracellular matrix (ECM). Aggregate-based culture systems allow a better mimicking of those aspects. Moreover, aggregate-based culture may avoid the need for adhesion matrices that are typically needed for cell adhesion, either in adherent or microcarrier cultures, hence reducing the number of components within the culture. To this end, single-cell inoculation and consequent aggregation of hPSCs has already been reported by several groups, under static conditions [17] and in spinner flasks that promote hPSC long-term culture and cell expansion up to 6-fold in 4 days [18,19].

**Figure 2:**
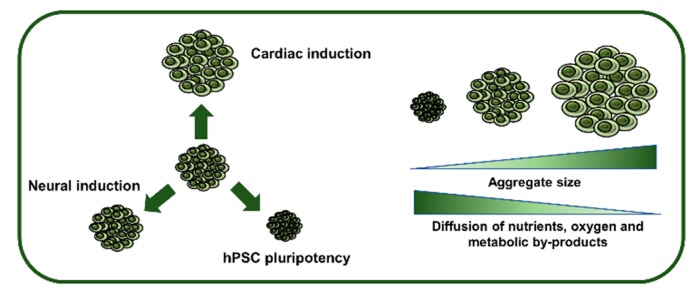
Influence of hPSC aggregate size on pluripotency maintenance, lineage specification and inner diffusion rates. The initial diameter of hPSC aggregates will determine the efficiency of induction into a specific lineage, as well as influencing the expansion capability. Furthermore, as aggregates increase in size, they are subjected to diffusional limitations that may cause necrotic zones in the inner part of the aggregates.

A major hurdle concerning hPSC commitment as 3D aggregates relies on the heterogeneity and disorganization that arises due to uncontrolled aggregation of cells, which in turn leads to poor efficiencies of differentiation. Control over aggregate diameter may be able to address this challenge, providing adequate and homogeneous cues within the aggregate and increasing culture uniformity [13] (Figure 2).

In order to fill this gap, various methodologies have already been developed, such as micropatterning techniques that have been employed to correlate aggregate diameter and its effect on pluripotency or differentiation trajectories [20]. Also, microwells are able to force cell aggregation and give rise to homogeneous populations [21], making them a powerful and relatively simple tool in creating size-controlled aggregates that can be efficiently directed towards a specific lineage, such as cardiomyocytes [22] and neural progenitors [23]. A recent publication highlights the use of chemical aggregation strategies, such as the use of dextran sulfate, that is able to promote hPSC aggregation while sustaining cell growth and pluripotency maintenance [24].

### 2.1. Control of Aggregate Size as a Tool to Direct hPSC to a Specific Lineage

In monolayer approaches, the initial density of the cells is important in order to control hPSC commitment [20]. For example, in neural commitment protocols using dual SMAD inhibition, starting with high cell densities results preferably in Pax6^+^ neuroepithelial cells, whereas if the protocol is started at lower cell densities the final outcome will consist of both central nervous system and neural crest precursor cells [25]. Likewise, commitment of hPSCs into brain microvascular-like endothelial cells has been shown to be dependent on cell seeding density in order to maximize the yield of blood-brain barrier cells [26].

Control over colony size is also crucial for pluripotency maintenance. For example, there are spatially dependent variations in Oct4 expression that can be linked to changes in local signaling activation. This has been correlated with pSmad1 expression, which is known to promote hPSC differentiation by antagonizing the expression of GDF3 [20]. Also, spatial patterning during embryonic development was shown to be regulated by Activin-Nodal, BMP and WNT signaling pathways [25,27,28]. In vitro, using BMP4 alone resulted in self-organization of hPSC colonies into layers radially arranged from the inside outwards, resembling the spatial organization of the three embryonic germ layers [7]. Additionally, it was verified that pluripotent cells were able to re-localize density-dependent receptors for BMP4 that will regulate self-organized differentiation patterns [29].

Translating this to 3D culture systems implies that control over cell numbers in each aggregate, and thus control over aggregate diameter, will influence the signaling pathways involved in the slender balance between pluripotency and differentiation [20]. For example, starting with micropatterned aggregates, it became possible to observe that smaller aggregates led to an endoderm-enriched population, whereas higher diameters tended to produce neural-enriched cells. Moreover, intermediate diameters, ranging 175–400 μm in diameter, led to high efficiencies in cardiac induction, up to 90% of hPSC-derived cardiomyocytes [11,30,31,32]. Additional studies further demonstrated that aggregates with an average diameter of 150 μm have greater potential to form neuroectodermal populations with Oct4^−^ Sox2^+^ phenotype than aggregates with smaller diameters [12,33].

Despite these studies, it is important to notice the diffusional limitations associated with 3D aggregates (Figure 2). These limitations can interfere with both expansion and differentiation of hPSCs [34]. For example, a study by Wu et al. demonstrated that in aggregates with a radius smaller than 100 µm, hypoxia was negligible [35], whereas in aggregates with more than 300 μm of diameter, a necrotic microenvironment may appear, possibly due to oxygen, nutrient and metabolic byproducts limitations [35,36]. In the end, even though control of the aggregate size and diameter can influence the trajectory of differentiation from the pluripotent state, the aggregate diameter should also be modulated according to the diffusional forces of metabolites, nutrients and oxygen.

**Figure 3:**
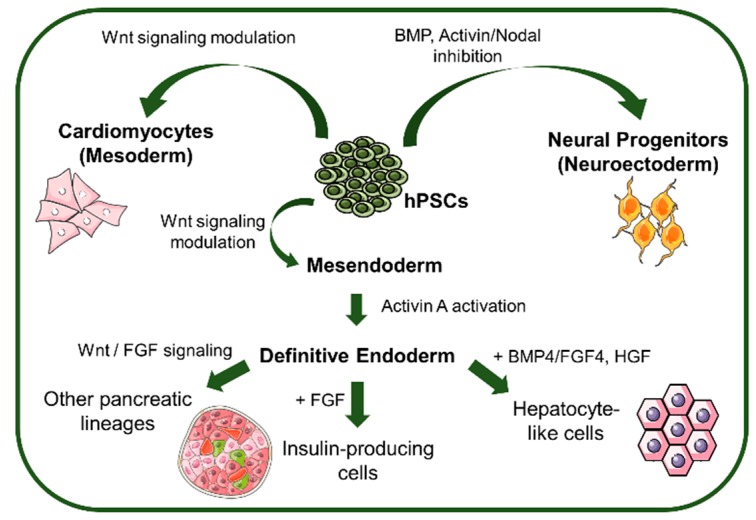
Signaling pathways and morphogens that control lineage specification from hPSCs. hPSCs can be differentiated towards the different lineages by BMP and Activin/Nodal inhibition (Neuroectoderm), Wnt signaling modulation (mesoderm and mesendoderm), and further differentiated to definitive endoderm by Activin A activation to generate pancreatic and hepatic lineage-derived cells. BMP: bone morphogenic protein, FGF: fibroblast growth factor, HGF: hepatoblast growth factor.

Derivation of hPSC into a specific lineage or cell type involves the regulation of different signaling pathways (Figure 3). In the next section, we analyze the influence of signaling pathways modulation and the influence of aggregate diameter towards specification of hPSC into neuroectoderm, mesoderm and endoderm.

### 2.2. Differentiation of Human Pluripotent Stem Cells Towards Neuroectoderm

Evidence that human embryonic stem cells (hESCs) could potentially be differentiated into specific mature cells of the nervous system and serve as an in vitro model for neurodevelopment was provided after obtaining cells from the three embryonic germ layers by spontaneous differentiation from hESCs [37]. The first generation of hESC-derived neural precursor cells (NPCs) exhibited properties comparable to primary neural stem cells (NSCs) in terms of expansion and differentiation capability, in a more immature stage than precursor cells derived from the fetal human brain [38]. These cells were obtained using heterogeneous embryoid bodies (EBs) containing cells from the three embryonic germ layers derived from the adherent monolayer of hESCs. From these aggregates it was possible to obtain proliferative NPCs that were able to further differentiate into neurons, astrocytes and oligodendrocytes [38,39]. Also, in some of these studies it was stated that the addition of fibroblast growth factor-2 (FGF-2) to the culture was able to give rise to large numbers of rosette-like structures, whereas the absence of this factor originated cell cultures without these organized colonies [38]. Furthermore, the dependence on FGF-2 was extended for survival and proliferation of NPCs, and the factor was added to posterior commitment protocols [40].

Knowledge and control over signaling pathways governing pluripotency maintenance and directed hPSC differentiation has led posteriorly to even more defined neural commitment protocols [41]. TFG-β superfamily plays an important role either in pluripotency maintenance or in differentiation. In the specific case of differentiation into the neural lineage, BMP and Activin/Nodal signaling inhibition are crucial. Addition of Noggin, a BMP-4 antagonist, to adherent and embryoid body (EB) culture gave rise to increased expression of several NPC markers [40,42]. Other antagonists, such as dorsomorphin and LDN-193189, a dorsomorphin analogue, can be used to inhibit BMP type I receptors ALK2 and ALK3 [43]. Still, the most common and efficient protocol for inducing hPSCs into NPCs consists in a simultaneous inhibition of both BMP and Activin/Nodal signaling pathways. This double inhibition, commonly known as dual SMAD inhibition, directs cells towards neuroectoderm while blocking signaling for trophectoderm, mesendoderm and endoderm [25,44]. Still, although the effect of soluble factors plays an important role in lineage specification, localized cell density can be considered another important variable, as it also influences different signaling pathways [20]. In the case of neural induction from hPSCs, high-density culture conditions are prone to generate cells from the central nervous system (CNS), whereas lower cell densities tend to give rise to peripheral nervous system (PNS) components [25,45].

Still, despite this knowledge, there are few protocols describing the derivation of hPSC into the neural lineage under 3D suspension conditions. The first report of neural induction from hPSCs under 3D suspension conditions used Noggin to direct cells towards a neuroectodermal fate [46], with an efficiency of over 90% of PSA-NCAM^+^ cells after 4 weeks of neural induction. Recently, in a different report of neural induction of hPSCs as 3D suspension aggregates showed a more homogeneous expression of PAX6 when compared to 2D cultures [47]. Using optimized parameters for initial aggregate diameter and duration of neural induction, Miranda et al. described for the first time a scalable and optimized process using chemically-defined conditions for neural induction of hPSC in 3D conditions [12]. By generating aggregates with an approximate diameter of 140 µm, it was possible to generate more than 80% of Pax6-positive NPCs that retained their multipotent potential. Moreover, further optimization led to an integrated process that could generate more than 14 × 10^6^ NPCs in only 6 days, at a 30 mL scale using spinner flasks [48].

Although production of NPCs from hPSCs can be performed using these robust 3D aggregate-based methodologies, the process of terminal differentiation from a NPC stage is often performed in 2D culture systems [25,44], which may fail to recapitulate the tissue architecture. Therefore, 3D culture methodologies that are able to mimic the original regional and subtype specificity are needed to model normal neurodevelopment, as well as to model neurodegenerative diseases [49]. In fact, although 3D culture systems have been developed and applied into modeling Parkinson’s [49] and Alzheimer’s [50] diseases, such systems use human midbrain-derived neural precursor cells from fetal origin and prenatal cortical neurons, respectively, to generate the models. Interestingly, culture of NPCs as 3D suspension aggregates has led to an increase in expression of glial markers during terminal astrocyte differentiation [47].

### 2.3. Differentiation of Human Pluripotent Stem Cells Towards Cells of the Mesodermal Lineage

Heart disease is the primary cause of death worldwide, and several pre-clinical studies are currently testing hPSC-derived therapy in large animal models of heart disease, thus requiring the generation of high numbers of cells from the cardiac lineage [51,52]. In addition, cardiac toxicity is one of the main causes of drug failure, and therefore it is important to develop new methodologies that will allow a more efficient drug screening process [53]. The first report of derivation of cardiac precursor cells as suspension aggregates under chemically defined conditions was based on the modulation of the Wnt signaling pathway, and employed the use of BMP4, Activin A, FGF2, VEFG and DKK1 to either activate or inhibit this signaling pathway at specific time points [54]. Nevertheless, the efficiency of the differentiation was a limiting step, as only 40–50% of total cells expressed cardiomyocyte markers at the end of the differentiation [54]. This method was further improved by forced aggregation of hPSCs using V-shaped microwells, and optimized concentrations of mesodermal morphogens BMP4 and FGF2, polyvinyl alcohol, serum, and insulin, that led to up to 89% of cTnT^+^ cardiomyocytes under chemically-defined conditions [55]. Furthermore, whereas a beating phenotype appeared at day 14 using conditions described by Yang et al. [54], according to Burridge et al. [55] a beating phenotype was already visible by day 7 of cardiac induction.

Cardiac induction of hPSCs as 3D aggregates has also been performed using dynamic conditions. Rotary agitation after 3D suspension aggregates generation in microwells was able to produce cardiospheres with around 100% contractility [56]. Suspension-based systems have been reported to yield up to 50 million cardiomyocytes in 100 mL controlled bioreactor scale [57]. Moreover, it was demonstrated that higher yields could be obtained by using cyclic perfusion instead of batch feeding regimens [58]. Also, a recent work by Chen et al. reports an average yield of 90% for hPSC differentiation into cardiomyocytes with scale up to 1 L spinner cultures using suspension aggregates [30]. Fonoudi et al. demonstrated the importance of control over aggregate diameter at the start of the cardiac induction from hPSCs, proving that maximum cardiac commitment efficiency is attained when the initial hPSC aggregate diameter is approximately 175 µm [31].

### 2.4. Differentiation of Human Pluripotent Stem Cells into Cells of the Endoderm Lineage

Differentiation into endoderm is initiated with a first step where neuroectodermal fate is inhibited, mainly by BMP4, Activin A and Wnt signaling, promoting the formation of mesendoderm [59,60]. At this point, a thin balance between mesoderm and endoderm exists and, by activating Activin A signaling a FoxA2^+^ population of definitive endoderm arises [61,62]. Further differentiation can be directed to insulin-secreting β-cells or other pancreatic lineages by adding FGF [63] or stimulating Wnt and BMP4 signaling [64], respectively.

Directed differentiation of hPSC as 3D aggregates into endoderm has improved the efficiency, homogeneity and functionality of the resulting populations when compared to 2D approaches. Clusters of multiple β-cells have exhibited larger quantities of insulin release upon glucose stimulation than single cells [65] and the same pattern was detected when comparing 3D aggregates with a 2D monolayer culture [66]. Starting with 120 µm diameter hESC colonies, subsequent exposure of cells to keratinocyte growth factor has originated primitive gut tube-like cells with homogeneous expression of HNF1β. From here, these cells could be further differentiated into PDX1^+^/NKX6.1^+^ pancreatic endoderm either from exposure to additional factors or from mechanical dissociation that would lead to the generation of spheres with an average diameter of 100 µm [67]. Differentiation into definitive endoderm followed by differentiation into hepatocyte-like cells (HLCs) was also performed as 3D aggregates being determined that the optimal aggregate diameter to initiate endoderm differentiation is approximately 130 µm [11]. This culture system has led to the production of up to 2 × 10^8^ HLCs in a spinner flask that demonstrated the improvement of hepatic function after hepatic failure in animal models [68].

## 3. From Aggregates to Organoids: Building Complexity

Although progenitor populations may be useful in the study of the first steps of embryo development, complexity remains a key component. Organoids are defined as 3D cell cultures, derived from stem cells or progenitors, resembling an organ, implying the existence of organ-specific cell types and regions, the capacity to mimic specific cellular organization, and lastly the ability to replicate some functions of the original organ [69]. Both the size and morphology of the differentiated spheroid favor the passage from a spheroid state to a pre-organoid [70]. As proven from the development of a fully formed optic cup from hESC aggregates, 3D architecture of an organoid plays an important role in modeling in vivo organization [71]. Organoids from different lineages have been derived in the last few years, from brain [72] to lung [73], optic cup [71], and gut [74], among others. As formation of organoids has the ability to closely mimic in vivo development, they offer a new approach towards disease modeling and drug discovery, also representing an important tool in studying various genetic disorders. Additionally, tailored-drugs for specific cancer subtypes can be designed from pancreatic tumor organoids derived from patient biopsies or from organoids obtained from colorectal tumors [75,76].

A current limitation of organoid culture relies on their large-scale production, which can delay the application of this technology. The fact that existing organoid culture relies on the embedding on a support extracellular matrix limits potential for scale up, despite providing the necessary environment for organoid growth and maintenance [72]. A recent approach highlights the use of a capsule-based platform, containing a Matrigel core, to support organoid growth, and an alginate shell that will protect the core from the shear stress in stirred bioreactors [77].

Although organoids can be produced from different stem cell sources, we will focus on organoids generated from pluripotent stem cells in the following sections.

### 3.1. Brain Organoids

Lancaster et al. demonstrated that 3D aggregates of hiPSCs-derived neuroectoderm that were encapsulated and cultured in Matrigel inside a spinning bioreactor resulted in spontaneous development of the so-called cerebral organoids. This process was able to recapitulate human cortical neurogenesis and to create a patient-specific model of microcephaly [72].

Using brain organoids, a recent study demonstrated that the folding of the brain relies mainly on the cytoskeletal contraction at the center of the organoid, as well as on the nuclear expansion in the outside of the organoid [78]. Using a miniaturized spinning bioreactor, it was already possible to generate forebrain, midbrain and hypothalamus organoids from hiPSCs that recapitulate fundamental characteristics of specific regions of the brain [79]. This type of region-specific brain organoids presented an inside-out pattern of cortical neurogenesis resembling a cerebral cortex-like structure [72,80]. In another report, self-organized cerebellar tissue derived from hPSCs in 3D culture produced a polarized epithelium which gave rise to electrophysiologically active Purkinje cells [81].

Brain organoids have been widely used to establish promising platforms to model neurodevelopmental disorders arising either from genetic diseases or environmental exposure. Specifically, in 2016 during the Zika outbreak in Brazil, region-specific brain organoids were used to demonstrate that the virus was the cause of the microcephaly cases that started to arise, by disruption of the growth of neural progenitor cells [82,83]. Another application of brain organoids technology involves the study of the effects that occur upon exposure of the developing central nervous system to various substances. For example, exposure to nicotine during early stages of human brain development led to neurite outgrowth and abnormal neuronal differentiation, caused by impairment in neurogenesis [84].

A current limitation in brain organoid technology consists in the lack of vascular circulation and immune systems. Among other functions, vascularization is essential for the oxygenation and nutrient supply of the organoid [85]. In order to bypass this issue, co-cultures with vascular cells have been attempted, which resulted in the formation of tubular-like vascular networks within the organoids [86]. An alternative approach consisted in the engraftment of the organoids in a mouse brain, which was able to promote the generation of a functional vasculature [87].

### 3.2. Kidney Organoids

Generation of kidney organoids will allow the mimicking of the effect of the presence of metabolic by-products in the blood stream upon renal excretion, creating an in vitro system that will allow identification of possible renal failure complications upon drug exposure.

Organoids containing multiple kidney components have already been produced from hiPSCs using a single protocol that generates the equivalent of a functional human fetal kidney containing ureteric epithelium, nephron progenitors, stromal progenitors and epithelial cells [88]. This protocol involves a first step comprising Wnt signaling pathway modulation by using CHIR99021 to induce the posterior primitive streak. In a second stage, FGF-9 and heparin were added for intermediate mesoderm induction, and a pulse of CHIR99021 at day 7 of differentiation was added to promote nephrogenesis. Further maturation without growth factors produced functional kidney organoids at day 25 of differentiation. Nevertheless, organoids generated using this protocol may probably not entirely reproduce kidney function, as it fails to induce the branching of the ureteric tree of the progenitor niche, which would lead to the formation of collecting ducts. To complement this approach, an organoid consisting of a specific differentiation into ureteric buds and nephron progenitor cells was also described [89]. Importantly, upon transplantation into a host, kidney organoids were able to promote host-derived vascularization, which enhanced organoid survival [90].

### 3.3. Liver Organoids

In parallel with kidney, 3D liver organoids are in the spotlight of the pharmaceutical industry, as hepatotoxicity accounts for the major failures in drug development strategies [91,92].

Like most of the internal organs, the liver derives from the definitive endoderm, and through a stepwise approach, liver bud progenitors and HLC can be obtained in vitro from hPSCs [93]. Liver buds are condensed tissue masses delaminated from the foregut that is further vascularized by angioblasts or endothelial cells to promote self-organization that will lead to the generation of organized and complex architecture of the liver [94]. After derivation of hepatic endoderm from hPSCs, the combination with mesenchymal stem cells (MSCs) and human umbilical vein endothelial cells (HUVECs) has led to the assembly of stable liver buds [95]. Co-culture of hiPSC-derived hepatic endoderm, MSCs and endothelial cells also enhanced vascular network formation through VEGF-mediated signaling [96]. Moreover, when grafted into a chimaeric mouse model, the integrated tissue was rapidly vascularized and able to increase the survival rates [95]. Nevertheless, a recent approach has reported the generation of liver buds entirely from hPSCs [97]. Progenitors from the three-lineages were derived from hPSC and were able to self-organize into liver buds, demonstrating functionality both in vitro and in vivo. Importantly, liver-organoid maturation can be analyzed by the presence of key cytochrome P450 enzymes and their ability to metabolize drugs, and the effect of selected hepatotoxic drugs can be assessed in terms of cell viability and organoid morphology [5].

### 3.4. Gut Organoids

The establishment of intestinal organoid cultures from hPSCs embodies a great advance in the generation of a model of the human intestine [74,98,99]. Adult intestinal stem cells have been shown to form 3D organoids with crypt-villus architecture when cultured in vitro [100]. The resulting organoids also contain the different types of cells usually present in the intestine, including enterocytes, entero-endocrine cells, goblet cells, and Paneth cells.

In addition, intestinal organoids can also be derived from hPSCs by the self-assembly of endodermal cells, creating a structure that resembles the epithelial lumen and the adjacent tissue of the intestine [74]. These organoids can be originated first by activin-induced definitive endoderm formation, followed by the combined activity of Wnt3a and FGF, which will promote posterior endoderm patterning, whereas FGF4 alone can afterwards promote hindgut morphogenesis [74]. Upon injection into a live host, hPSC-derived organoids form mature structures of intestinal epithelium, which is supported by the host vasculature [101]. Although in the past a fully mature phenotype could only be obtained by engraftment into a host, a new approach has reported the generation of gut organoids that are able to fully mature in vitro [102].

Intestinal organoids have also been derived from colorectal cancer patients and, upon in vitro culture, were capable of producing cancer organoids that allow the characterization of their mutations [103]. Besides the accentuated differences between the numbers of somatic mutations that cancer organoids presented when compared to normal colorectal cells, there were also different responses to anticancer drugs.

## 4. Organ-on-a-Chip Devices: Towards the Development of Multi-Organoid Platforms

The efforts to create a platform that could fulfill the gap between in vitro testing and animal models has led to the development of organ-on-a-chip devices for drug screening applications. Organs-on-a-chip aim to combine the ability of recapitulating aspects of human physiology under a controlled environment that could be conjugated with microfluidics to engineer a platform that can revolutionize drug development and safety, as well as clinical studies [104]. Recent reviews have summarized the advantages of building microfluidic platforms of organs-on-a-chip that could be used towards engineering human tissues to accelerate drug development and safety assessment [105,106].

It has been over a decade since the first attempts to combine different tissues within a single platform were performed [107]. After these first experiments, a number of different devices have been created using tissues obtained from commercially available cell lines and primary cells [5,108,109], Multi-organ platforms are especially important in drug discovery applications, as one must consider that a drug is never delivered only to a specific site, but instead it is metabolized and runs through various organs before completing its final action. Therefore, interactions of drugs and their metabolites with the different parts of an organism are as important as the action that the drug will perform in its final target site.

There are some technical challenges associated with the creation of multi-organ platforms, such as (i) fabrication of a platform that can withstand prolonged culture times of the different organ tissues; (ii) the use of a universal culture medium that can support different cell types; and (iii) the mimicking of the representation of the in vivo ratios of each type of organ [110,111]. In fact, highly important parameters in designing an organ-on-a-chip device are the ratio of the mass of the different organoids to accurately mimic the most suitable physiological conditions, in combination with respect for the cellular heterogeneity ratios of the organ [109].

One good example of an organ-on-a-chip device containing different organ representatives consists of a microfluidic setup comprising four connected organ tissues—intestine, liver, skin and kidney—allowing prolonged culture periods while maintaining a controlled microenvironment [108]. Yet, this chip comprises organ tissues mostly at a 2D level, and only liver buds are represented as 3D structures. Importantly, cell sources to build this system come from primary cells or from commercially available cell lines, which from a drug discovery point of view may function but fail to address a personalized medicine approach.

The most advanced culture models consist in a chip platform that contains three different modules, comprising liver, heart and lung organoids [5]. This microchip has been used to test the effect of different drugs on each organoid individually as well as their effect on the system as a whole. For that, there are sensors and cameras incorporated within each module that allow the assessment of the cardiac beat rate of the heart organoids, the culture medium analysis of albumin, urea, lactate dehydrogenase and alfa-glutathione-*S*-transferase for the liver organoids, and electrochemical sensors to detect ion channels responses for the lung organoid.

Nevertheless, most of the abovementioned systems rely on the use of commercial or primary cell sources to generate organoids capable of mimicking the original tissue. Primary cells are able to maintain similar levels of metabolic and functional properties as the original tissues. Nevertheless, their accessibility restricts their use, as well as the loss of metabolic function when cultured in vitro for long periods of time [112]. On the other hand, immortalized cell lines often fail to represent the original tissues, and tend to accumulate karyotypic abnormalities that may compromise the results [113]. In contrast, hiPSC-derived cells hold the promise to replicate the function of the representative tissue. For example, hPSC-derived hepatocytes demonstrate CYP450 metabolism and produce albumin, though they might display lower levels of enzymatic activity [114]. Therefore, for future applications, the gold standard would comprise a microchip with different types of organoids directly differentiated from hiPSCs. This would enable the generation of patient-specific platforms from an unlimited source of cells that could be used for personalized medicine applications.

## 5. Conclusions and Future Challenges

The unique properties of human pluripotent stem cells have contributed to their growing significance in fields such as regenerative medicine, disease modeling and drug discovery. Frequently, in vitro directed hPSC differentiation protocols are based on adherent cultures that often fail to mimic in vivo development in terms of spatial organization. However, 3D structure conferred by aggregate culture allows a better mimicking of in vivo conditions, including appropriate presentation of patterning signals that influence self-organization [71,72,81,115]. Hence, different methodologies can be used to produce 3D aggregates, ranging from spontaneous aggregation in suspension cultures to microwells. Choosing an adequate system for the desired application is very important: microwells are useful for high-throughput analysis, whereas spontaneous aggregation under dynamic conditions is valuable for scale-up processes. Moreover, control over the size of the aggregates can help to manipulate cell fate, as different initial diameters may influence the preferential differentiation pathway of cells. Scaling-up hPSC culture can also be facilitated using 3D aggregates, circumventing the need for adhesion surfaces and potentially reducing overall costs.

Importantly, as it was highlighted in this review, 3D culture of hPSC is a promising platform to recreate in vivo microenvironments, and by controlling the diverse parameters, can be used to efficiently differentiate hPSC into tissue precursors and further down, into organoids of specific cell lineages mimicking the structure and function of the respective organ. One important limitation plaguing the application of this technology, as the size of the organoids increases, the issue of tissue necrosis caused by the lack of diffusion of oxygen and soluble factors needs to be addressed, probably by activating angiogenic pathways that will lead to vascularized organoids. In particular, this challenge was partially bypassed by the creation of vascularized liver buds from hiPSCs [95].

In this review, we anticipate the potential of culturing distinct hPSC-derived organoids in organ-on-a-chip devices. Despite recent developments, the full potential of multi-organ platforms is still hampered by several challenges. Among them, we highlight the need for universal culture media that will provide optimal conditions for culturing different types of organoids, each one containing a wide range of cell types that need specific physiochemical cues. In the future, the overall goal will be the development of microchips containing different types of hPSC-derived organoids representing the structure and function of multiple organ systems for screening the effects of drugs in more in vivo-like settings.

## Figures and Tables

**Table 1:** Comparison between animal models and in vitro 2D and organoid cultures derived from human induced pluripotent stem cells (hiPSCs) as tissue models for drug discovery. Legend: • low, •• medium, ••• high.

	Representation of Native Human Tissue	Ethical Issues	Cost	Maintenance	Throughput	Whole Body Assay
Animal models	**•**	**••**	**•••**	**••**	**•**	**•••**
2D	**•**	**•**	**•**	**•••**	**••**	**•**
Organoids	**•••**	**•**	**•**	**•••**	**••**	**•**
